# Historic and Prehistoric Epidemics: An Overview of Sources Available for the Study of Ancient Pathogens

**DOI:** 10.3390/epidemiologia3040034

**Published:** 2022-10-07

**Authors:** Antoinette C. van der Kuyl

**Affiliations:** 1 Laboratory of Experimental Virology, Department of Medical Microbiology and Infection Prevention, Amsterdam UMC, University of Amsterdam, Meibergdreef 9, 1105 AZ Amsterdam, The Netherlands; a.c.vanderkuyl@amsterdamumc.nl; Tel.: +31-205-666-778; 2 Amsterdam Institute for Infection and Immunity, 1100 DD Amsterdam, The Netherlands

**Keywords:** prehistory, history, archeobiology, paleovirology, EVEs, ancient DNA, paleogenomics, pathology collections, historic publications

## Abstract

Since life on earth developed, parasitic microbes have thrived. Increases in host numbers, or the conquest of a new species, provide an opportunity for such a pathogen to enjoy, before host defense systems kick in, a similar upsurge in reproduction. Outbreaks, caused by “endemic” pathogens, and epidemics, caused by “novel” pathogens, have thus been creating chaos and destruction since prehistorical times. To study such (pre)historic epidemics, recent advances in the ancient DNA field, applied to both archeological and historical remains, have helped tremendously to elucidate the evolutionary trajectory of pathogens. These studies have offered new and unexpected insights into the evolution of, for instance, smallpox virus, hepatitis B virus, and the plague-causing bacterium *Yersinia pestis*. Furthermore, burial patterns and historical publications can help in tracking down ancient pathogens. Another source of information is our genome, where selective sweeps in immune-related genes relate to past pathogen attacks, while multiple viruses have left their genomes behind for us to study. This review will discuss the sources available to investigate (pre)historic diseases, as molecular knowledge of historic and prehistoric pathogens may help us understand the past and the present, and prepare us for future epidemics.

## 1. Introduction

The most obvious evidence of parasites preying on earthly life forms can be found in our chromosomes, were various viruses have left their genomes as a testimony of past infections [1]. Proof of the infectivity of these so-called endogenous viruses can be found in recent and/or ongoing invasions of the host germ line, for instance in mice, cats, and koalas, as well as in ancient between-host viral transmission (reviewed in [2,3,4]), and the resurrection of endogenous human retroviruses to their infectious counterparts [5,6]. In addition, selection in immunity- and infection-related host genes illustrates the ongoing battle with pathogens, although of course here the causative pathogen is likely to remain anonymous.

Ancient burial patterns can also display evidence of past infectious disease outbreaks, especially when there is no evidence of external violence to the remains. When many die around the same time, people are either carefully buried together in the same grave, or bodies are hastily thrown into quickly dug graves. Likewise, since historic times, our ancestors have put pen to paper to describe epidemics affecting society, for instance, the ancient Greeks described the multiple plagues of their times, while an inscription of the local word for pestilence on 1338–1339 CE century tomb stones, in what is now Kyrgyzstan, pointed to the likelihood of plague victims being buried there [7]. Another example can be found in the relatively accurate descriptions of a smallpox-like disease in Chinese texts dating to the Sui and Tang dynasties, 581–907 CE [8]. Such information can be important to understand past epidemics, or to identify places to hunt for ancient pathogens, but the sources themselves are less helpful in identifying the infectious causes, as they rely heavily on clinical symptoms, which are rarely diagnostic, and certainly do not provide evidence for the involvement of a particular variant.

Although all of the above information is important for understanding our past, it was not until the development of ancient DNA techniques, and the understanding of DNA degradation mechanisms, that the actual detection of ancient pathogens in (pre)historical samples became feasible. Some studies focus on PCR detection of a specific pathogen in a historical sample, guided by clinical symptoms such as skin eruptions in a mummy suggestive of smallpox [9], mass burials coinciding with described plague epidemics [10], the plague of Athens [11], or the likelihood of infection in victims interred during the 1918 influenza pandemic [12]. Nowadays, modern sequencing techniques enable the retrieval of partial or complete ancient pathogen genomes (reviewed in [13]). In addition, genomic shotgun data often contain pathogen DNA, enabling identification and assembly of viral and bacterial genomes, as exemplified, for instance, by hepatitis B virus (HBV) DNA in Neolithic, Bronze Age, and medieval sequence samples [14,15,16]. 

This review will emphasize the importance of studying past pathogens by giving an overview of the sources available to researchers for the study of historic and prehistoric diseases, and their associated epidemics, rather than providing an exhaustive overview of ancient pathogen studies. Coping with the struggles of retrieving and authenticating ancient DNA, as well as with sequence analysis of ancient microbial genomes, have been the subjects of some excellent, recent reviews [13,17,18,19].

## 2. Materials and Methods

The PubMed database [20], Google Scholar [21], and Google Books [22] were manually searched with appropriate terms such as ”ancient DNA”, “archeovirology”, “endogenous retrovirus”, “EVE”, and “paleogenomics”, and eventually a second term appropriate to the topic, such as “pathogen”, “historic”, “prehistoric”, “evolution”, “dating”, etc. Abstracts were inspected for relevance; from the selected abstracts, only those of which a full-length publication could be retrieved were read and included. In addition, references in the retrieved papers were inspected and used, when appropriate. The purpose of the current paper is to provide a comprehensive overview of the field, not an exhaustive review, so not all papers retrieved will be discussed.

## 3. Results

A strict definition of the terms “pandemic“, “epidemic“, and “outbreak“ does not exist, but a more general description of all three would be “a sudden increase in disease cases above the normal, endemic, disease burden in a certain area, community or population“, either caused by a novel pathogen, an increased transmissibility or virulence of an established pathogen or any other important variation in the behavior of the pathogen, or an increase in host susceptibility or exposure [23]. To reduce confusion, the term “epidemic” will be used here as a synonym for the historical concepts of “plague“ and “pestilence“, and as a synonym of the term “outbreak“. The word plague, from the Latin *plaga*, meaning “stroke, or wound”, a common term for an “epidemic with many deaths”, was from the beginning of the 17th century CE increasingly used to specifically describe *Yersinia pestis* infections [24]. It must be noted that not all ancient pathogens are thus epidemic pathogens in a strict sense, and that epidemic pathogens can persist and thus become endemic, while endemic pathogens are often able to cause, small, outbreaks. Examples of the latter would be influenza virus, human parvovirus B19, measles virus, and *Yersinia pestis* in medieval and early modern times. Twenty-first century endemic pathogens, which are widespread, were introduced into the human population a long time ago, and that cause few or no outbreaks at present, would for instance be *Plasmodium* species (causing malaria), intestinal parasites, Mycobacteria (causing tuberculosis and leprosy), *Helicobacter pylori*, HBV, and herpesviruses. For an overview of the sources of ancient pathogens discussed below, see Table 1. 

### 3.1. Contemporary Genomic DNA as a Source of Ancient Pathogens and Epidemics

Genomes from living organisms contain the remnants of the ongoing battle between pathogens and hosts, both as integrated viruses and as selective sweeps in immune-related genes. To look for an easy-accessible source of the most ancient pathogens, all we have to do to study those carefully propagated leftovers of the past is to sequence and analyze the corresponding DNA stretches in modern chromosomes. For the older viruses, those invading host genomes over 50–100 million years ago (mya), most of their genomes are likely to be useful only in retracing evolution by descent, rather than informing us of putative transmissions or epidemics caused by those parasites. However, when sufficient endogenous viral elements (EVEs) are present in a species, or in related species to reconstruct a detailed evolutionary history, their past transmission patterns may become visible, especially since more and more complete genomes are being sequenced and becoming accessible from public databases. Comparison of simian endogenous retrovirus (SERV) genomes in Old World monkey species, for instance, showed that this virus was spreading among African and Asian monkeys less than 8 million years ago (mya) [3], while tracking down single-stranded RNA virus-related integrations suggested that around 40 mya, ancestral mammals suffered from a heavy load of borna- and filovirus infections [25,26]. Of course, virulence and pathology cannot be estimated from endogenous viral genomes alone, although similarity to modern pathogens could suggest this. In theory, transmission of ancient viruses could have resulted in little or no morbidity and mortality, which would then disqualify them from being the cause of *bona fide* epidemics. However, witnessing recent, successful novel introductions of microbes into human populations or domestic animal populations provides ample evidence of those microorganisms being real pathogens, with at least part of a population experiencing disease. It is not likely that this would have been different in the past. 

Modern genomes can likewise show evidence of ancient battles by rapid local evolution, or by selection, positive or negative, of existing alleles from genes involved in either immunity or in the replication cycle of ancient parasites. Examples are, for instance, the acquisition and fast evolution of the APOBEC3G gene in primates (reviewed in [27]), and the dominance of a dysfunctional Toll-like receptor 1 variant associated with protection against leprosy in people of European descent [28]. A 32-base pair deletion in the CCR5 gene, already found in prehistoric Europeans, has likewise been attributed to such a selective sweep, although the causative pathogen remains a mystery, after bubonic plague was ruled out [29,30,31]. However, selection of certain immunity-related alleles, such as those encoding Ficolin-2, NLRP14, and HLA-DRB1*13, involved in the response to pathogenic bacteria, was obvious from a genetic comparison of thirty-six 16th century plague victims buried in Ellwangen, Germany, with modern inhabitants of that city [32]. It is therefore likely that the plague pandemics did influence the genetic make-up of surviving populations. High carrier frequencies for pyrin—an inflammasome protein—mutations seen in Mediterranean populations have indeed been associated with resistance to *Y. pestis* [33]. Other allelic selections associated with epidemic pathogens are, for instance, the highly prevalent FUT2 (an α(1,2) fucosyltransferase involved in H type 1 blood group antigen synthesis) non-secretor alleles with resistance to noro- and rotavirus infections [34,35,36], IFITM3 alleles (encoding an interferon-inducible transmembrane protein) with influenza morbidity and mortality [37], DARC gene (encoding a chemokine receptor) variation with malaria resistance [38], a tyrosine kinase 2 (TYK2) polymorphism with clinical TB [39], and IFNL4 (encoding an antiviral interferon) pseudogenization with hepatitis C virus clearance [40]. In a Bangladeshi population, multiple genomic regions were found to be selected in association with cholera susceptibility [41]. These stretches, for instance, encode potassium channel genes, nuclear factor κB (NF-κB) signaling components, and proteins of the NF-κB/inflammasome-dependent pathway [41]. Glucose-6-phosphate dehydrogenase (G6PD) deficiency, and beta thalassemia- and sickle-cell disease inducing mutations, prevalent in Africa and the Mediterranean, are all linked to malaria resistance (reviewed in [42]). A more complete overview of pathogen-induced selection on human genetics can be found in [43,44].

Due to improved technology and decreased costs, the number of complete genomes is rapidly increasing, which will greatly enhance our knowledge of ancient host-microbe battles.

### 3.2. Archeological and Historical Sites as Sources of Ancient Pathogens and Epidemics

Archeological and historical burial sites can be good sources to look for patterns of epidemics and their causative pathogens in the distant past. For instance, a sudden increase in the number of individuals interred, or a high frequency of adolescents and young adults among the deceased; abnormal burials, as demonstrated by multiple or mass graves; bodies stocked in layers; the use of lime; un-coffined burials; and/or other sloppy burial practices can all point to a major disaster, possibly in the form of an epidemic, especially when there is no evidence of physical trauma [45,46]. Such locations have been termed “mortality crisis” graves [47]. Grave artifacts, such as coins and clothing, ^14^C dating, and written evidence in the form of tombstones, or parish records, can be used to date the burial grounds. For instance, three mass burials discovered in the center of Ellwangen, Germany, concerning a total of 101 skeletal remains buried close together, with other evidence, such as the majority being children, a characteristic of later plague epidemics, suggested that the site harbored 16th–17th century plague casualties [48]. Indeed, the presence of *Yersinia pestis* DNA was confirmed in dental pulp extracts [48]. The remains were also used to investigate the genetic variation of the victims [32]. In a plethora of other studies, teeth and bone samples from putative 14th–18th plague “pits” in many locations in Eurasia have been confirmed to contain *Y. pestis* DNA [7,47,48,49,50,51]. From the 17th century onwards, direct written evidence of plague epidemics and dedicated graveyards is common [7,47,51]. Retrieval of *Y. pestis* DNA from victims of earlier epidemics, such as the 6th century “Justinian plague”, has also relied on evidence for local mortality crises, such as when a “striking number” of double and multiple burials was seen in a grave-yard in Bavaria, Germany; when collective burials were found in Valencia, Spain; and when an existing ditch was used as an emergency cemetery near Bourges in France [52,53]. In contrast, burial patterns in Neolithic and Bronze Age graves have not been found to be useful in this regard, likely due to a lower population density, so that samples from those periods are usually tested at random for pathogens [13,54,55,56]. Similarly, in Africa, where mass burial sites, which could represent plague pits, have not yet been found, it has been speculated that two- to fourfold simultaneous interments may already be indicative of epidemic disaster, as could be a population reduction resulting in abandonment of settlements, infrastructure decay, or fluctuations in industrial activities [57,58]. Of course, such developments could easily be attributed to other causes, such as famine or climatic disaster, but epidemics should certainly be considered when no war or other type of major violence is present. 

In contrast to plague emergency burials, for other common medieval diseases, such as leprosy, dedicated leprosaria had been erected from medieval times onwards to house and isolate the large numbers of patients. Leprosy, or Hansen’s disease, is caused by *Mycobacterium leprae*. It runs a chronic, slow course, leaving sufficient time to bury the dead peacefully in cemeteries within the institution. Ancient DNA analysis of remains from medieval European leprosaria, resulted in the retrieval of partial and complete genomes of *M. leprae*, with a surprisingly high genetic diversity [59,60,61]. Complete *M. leprae* genomes were likewise retrieved from bones collected from a 10th-12th century British leprosarium [62], confirming that dedicated hospital sites are fertile hunting grounds for research on pathogen evolution. Evidence from ancient remains of another chronic disease, namely tuberculosis (TB), which is caused by another Mycobacterial species complex, *Mycobacterium tuberculosis*, also shows the substantial worldwide genetic diversity of this ancient pathogen in the past, compared with modern isolates [63,64,65,66]. Detection of *M. tuberculosis* DNA and mycolic acids from a double burial of a ~25-year old female and a ~12-month old infant dating to around 9000 years BCE in what is now Israel, demonstrated that infection with the bacterium could be rapidly fatal in the past [67]. The child’s skeleton showed extensive bone pathology indicative of disseminated neonatal TB, to which young children are highly susceptible when infected by someone with contagious pulmonary TB [67]. 

Other locations where emergency burials might be expected are battlefields and sites of temporary army settlements. There, death from infectious diseases is equally likely as dying from violence, as military camps are, and have been, sites of mass crowding, stress, poor hygiene, poor nutrition, as well as the gathering of soldiers from different backgrounds. In the absence of battle injuries, mass graves near army quarters may be an indication of an epidemic of some sort. Local archives, for instance, suggested that mass graves excavated near Vilnius, Lithuania, contained the remains of soldiers from Napoleon’s Great Army, retreating from Moscow in December 1812 [68]. The deceased had been quickly interred, usually before rigor mortis had set in, and were buried close to each other. DNA from the louse-borne pathogens *Bartonella quintana*, the causative agent of trench fever, and of typhus-inducing *Rickettsia prowazekii*, was found in up to one-third of the soldiers, with the first also being found in three lice samples, indicating that these diseases had had a great impact on those surviving the combat of Napoleon’s Russian campaign [68]. DNA of the above mentioned louse-borne pathogens was also found in soldiers buried in a mass grave near the besieged city of Douai in northern France, dating to 1710–1712 CE [69], while *B. quintana* and also *Y. pestis* DNA were retrieved from two French multiple-burial sites in Bondy, dating to the 11th–15th centuries CE, and putatively containing plague victims buried closely together without coffins [70]. Finding ancient DNA from louse-borne diseases in mass graves together with *Y. pestis* DNA has led to the hypothesis that in medieval times, the spread of human louse- and not rat flea-borne *Y. pestis* was the source of the plague epidemics [71]. DNA from another louse-borne pathogen, *Borrelia recurrentis*, the bacterial cause of relapsing fever, was detected in dental pulp from an individual buried in a 15th century CE double grave in Oslo, Norway [72], while paleo-serology was able to identify seven *Borrelia* infections (most likely *B. recurrentis*, as *Borrelia* spp. inducing Lyme disease do not cause epidemics) and one *B. quintana* seropositive tooth from a 16th–17th century military garrison cemetery in Auxi-le-Château, France [73], again indicating that louse-borne pathogens were common in historical Europe. 

Lack of war injuries, a contemporary chronicle of a “pestilence” suffered by both besiegers and besieged during an attack on Barcelona, Spain, in 1652, and the presence of both shallow graves, as well as large pits with dozens hastily buried with their boots still on, led to the discovery of *Salmonella enterica* Paratyphi C DNA in one of the victims, suggesting enteric fever could have been the cause of the documented pestilence, and not *Yersinia pestis*, as previously assumed [74]. A related *S. enterica* Paratyphi C strain had earlier been identified as the likely cause of an epidemic in colonial Mexico during 1545–1550 CE, where a local cemetery witnessed “catastrophic mortality”, demonstrated by stacks of corpses, and an overrepresentation of adolescents and young adults [75,76]. Historical accounts from medieval Lübeck, now in Germany, recorded two epidemics in 1350 CE and 1359 CE, which were likely Black Death outbreaks, together with at least four other unspecified pestilences occurring there during the 14th century [77]. Subsequent sequence analysis of the remains found in two multiple burial pits next to the, still in use, Heiligen Geist Hospital, showed *S. enterica* Paratyphi C in eight of them, implicating enteric fever as the cause of death [77]. Interestingly, no *S. enterica* Paratyphi C DNA was found in teeth and bones from two adjoining mass burial graves, which are supposed to be slightly older, neither were reads corresponding to *Y. pestis* DNA, leaving the question open as to what caused the death of those victims [77]. Virulent precursor strains to the modern enteric fever-causing Salmonellae may already have been involved in ancient epidemics, as the detection of DNA of the so-called *S. enterica* Para C lineage was found in 3000 BCE Bronze Age remains from multiple simultaneous burials, mainly of infants, children, and young adults, in a cemetery near Xinjiang, China [78]. The first documented case of *S. enterica* Paratyphi C in Europe dates to 800 years ago, when a young female, who most likely died of enteric fever, was buried in Norway [79]. Her single burial in cemetery near a church, however, did not suggest an ongoing epidemic.

Therefore, investigation of historical sites, guided by abnormal burial patterns is a fruitful method of biological sample selection, with the associated benefit that there is already support for the epidemic potential of any pathogen retrieved. A disadvantage is that the mortality crises used to guide ancient DNA research are mainly apparent in densely populated regions, implying a focus on more recent times. However, in the permafrost in northeastern Siberia, an unusual burial of five bodies in a single grave drew the attention of archeologists, as individual interment was the standard practice in late 17th–early 18th century Yakutia [80]. Variola virus (VARV) DNA fragments could be retrieved from one of the corpses, suggesting that these people had died from smallpox, and that the simultaneous deaths, indeed, did cause some sort of a crisis in their community. 

### 3.3. Archeological and Historical Samples as a Source of Ancient Pathogens

The emergence of ancient DNA research several decades ago, saw molecular biologists begging for pieces of valuable or, at least in the beginning, not so valuable, archeological and historical specimens, to serve as a possible source of ancient DNA. Pathogen DNA retrieved from such unique objects cannot on its own cover epidemics, but it can significantly contribute to our knowledge of the earliest features and evolution of microbes known to be able to cause epidemics. (Electron) microscopy, targeted PCR amplification, employing microarrays, and microbial reads being present as a byproduct of deep sequencing have all been used to detect ancient microbes or their DNA in (pre)historic samples. Identification of a pathogen can already give valuable information on its appearance in history, but to learn something about the virulence and genetic variation, more information is needed. Fortunately, with the advance of ancient DNA techniques, generating complete or near-complete ancient pathogen genomes is increasingly feasible (reviewed in [13]). The results from such studies are already changing our view on ancient pathogens and epidemics. For instance, using multiple specimens collected from variable periods and geography, it was shown for *Y. pestis*, *M. leprae*, *M. tuberculosis*, VARV, and HBV that the genetic variation of these pathogens was much larger in the past. Another interesting observation was the presence of multiple pathogens in individuals, highlighting the infectious disease burden that plagued our ancestors. Examples here are the presence of both *Y. pestis* and *Haemophilus influenzae* genomes in the dental pulp of a 6th century English child, where the observed extensive scarring of the bones could be explained by the invasive *H. influenzae* infection causing septic arthritis; and the finding of both *Y. pestis* and *T. pallidum pertenue* DNA in a 15-16th century CE Lithuanian plague victim [81,82].

Variant strains of *Treponema pallidum*, the bacterial cause of syphilis, yaws, and bejel (endemic syphilis), depending on the subspecies, were shown to be widespread in early modern times in both Europe and Colonial Mexico [83,84]. As a result, the mystery as to what was the cause of a late 15th century epidemic in Europe, described in 16th century medical literature as an acute, severe venereal disease, has not yet been solved [82,85,86]. Was it due to the introduction of *T. pallidum pallidum* from the New World or elsewhere, or due to the introduction of a novel strain of that bacterium, or was the disease not syphilis at all, as until the 19th century, the various venereal diseases were often confused with each other and with leprosy, while the symptoms described in those early manuscripts are very different from the chronic, relatively mild disease that syphilis soon became [84,86,87,88]. As *T. pallidum* sequences have been successfully obtained from both bone and teeth, it is likely that in the near future more samples will be located, so that further bacterial genomes can be added to help solve the puzzle. In particular, analyzing a well-dated sample from the early years of the epidemic, 1495–1545 CE, would help significantly. 

Interesting results were also obtained after sequencing ancient VARV, the causative agent of smallpox, which was shown to have arisen as late as the 16th or 17th century, as VARV isolated from earlier samples, dating to the ~600–1050 CE, belonged to a different viral clade, which was not a direct ancestor of the later VARV [89]. It is not clear whether or not this Viking-age VARV carried a similar lethality to smallpox, as its genome did not have the gene reductions associated with virulence in orthopoxviruses. However, as VARV does not cause chronic infections, it was suggested that the victims positive for VARV DNA had died during an acute infection, which would be indicative of a fatal disease associated with the virus [89]. VARV sequences detected in lung tissue from one in five family members buried together in a single emergency grave in the Siberian permafrost in the late 17th or early 18th century, suggest that by then the virus was able to cause severe disease, although skin pustules were not observed, and phylogenetic analysis suggested that the virus belonged to a progenitor strain to modern VARV [80]. For HBV, many more viral genotypes were found in Neolithic and medieval samples than are currently known [14,15,16]. In addition, the modern geographical distribution of HBV genotypes was found to be significantly different from that in the past [15,16]. Thus, this currently widespread virus, similarly to the extinct VARV, also had a much broader distribution and genetic variation than estimated from modern samples. 

Another virus with a worldwide distribution and a long-term association with humans is parvovirus B19 (B19V), a highly-infectious, blood-borne DNA virus capable of inducing multiple severe pathological conditions, besides being the agent of the fifth disease in children. Regional prevalence of B19V varies, and outbreaks of the virus are common. After having first been identified in 70-year old bones from Finland [90], B19V DNA was also found in ancient dental and skeletal remains from Eurasia and Greenland dating to 500–6900 years ago, confirming a lengthy presence in humans [91]. Three B19V genomes, reconstructed from dental remains present in mass graves near a colonial hospital in Mexico City, which had been used to bury epidemic victims during the 16th–18th centuries, illustrate an association of the virus with deadly epidemics in the past [92].

Human samples used to isolate ancient pathogen DNA range from bone, teeth (both dental pulp and dental plaque), dried/mummified skin and other (wet, fixed, or frozen) tissue samples, feces, and even chewed birch pitch. Gut contents and paleofeces, also known as coprolites, have been used to detect both DNA and microscopic evidence of intestinal parasite infections; for instance, *Ascaris* eggs were found in stool from a latrine, and in mummies dating to 14th century Europe and 16th–17th century South Korea [93,94]. Helminth eggs and DNA were even detected in sediment samples taken from under the pelvic area of skeletons [95,96,97,98,99,100]. In addition, paleofeces can be used to reconstruct the intestinal microbiome and the intestinal virome, while dental plaque can offer insights into the oral microbiome [101,102,103,104,105,106,107,108]. A little studied, but interesting, source of ancient pathogens could possibly be found in collections of ancient Egyptian canopy jar holders, in which internal organs, and later even heads, from the deceased were preserved during the mummification process [109]. Canopy jars, containing liver, lung, stomach, and intestine, were part of the ancient Egyptian funerary practice from approximately 2600 BCE until the Roman period [109]. Indeed, calcified *Schistosoma haematobium* eggs have been identified in mummy liver, pointing to a long-term presence of bilharzia in Egypt [109,110]. 

With all samples, showing the presence of pathogens is the first step, but complete or near-complete microbial genomic information, ideally from multiple samples, is needed to understand the pathogen’s evolution and role in ancient epidemics. Especially for bacteria, virulence genes should be identified, as bacteria without those are generally benign, so that the presence of a particular species does not necessarily indicate pathogenicity. For instance, the acquisition of virulence factors plays an important role in the evolution of *Y. pestis*; developments that have been retraced, helped by ancient pathogen DNA studies. Investigation of bacterial genomes from various archeological sites showed that the common ancestor of historic and current Black Death-causing *Y. pestis* strain originated from 14th century Central Asia, in what is now Kyrgyzstan [7]. In this region, around 1338–1339 CE, a low-complexity variant equipped with a few extra virulence factors, as compared to its Neolithic predecessors, entered human society, never to leave again [7,47,54,55,111].

### 3.4. Pathology Collections as a Source of Historical Pathogens

Pathology collections, containing the actual body parts, tissue, or blood samples of those who lived less than a few centuries ago, the oldest of which can be found in museums, and the more recent in biobanks, medical archives, or hospital freezers, are a valuable source when looking for historical pathogens in “wet” samples. As such collections are likely to contain only the most interesting specimens, it is improbable that epidemics could be reconstructed by analyzing their samples, yet information regarding the genetic make-up of an example microbe from a documented outbreak can be very valuable. For instance, analysis of an intestinal sample collected from a 1849 victim of the second cholera pandemic showed that *Vibrio cholerae* in the specimen indeed belonged to, as assumed, the classical O1 biotype, which was replaced in the 20th century by the El Tor O1 biotype [112]. Both a plasma sample and a lymph node biopsy from 1959 and 1960, respectively, stored in what is now the Democratic Republic of the Congo, have been used to amplify human immunodeficiency virus type 1 (HIV-1) genomic fragments [113,114]. The results indicated that the virus was not only definitely present by then, but had also diversified into the genetic subtypes seen at present, implying that its origin in humans dates back to at least the early 20th century. Formalin-fixed, paraffin-embedded lung tissue from victims of the 1918 influenza virus pandemic, as well as lung tissue biopsies taken from those buried in permafrost, have likewise suggested the probable origin (novel re-assortment of avian and human flu viruses), characteristics (hemagglutinin (HA) and polymerase genes contributed to its virulence), and genetic variation (evidence for early adaptation to humans in HA) from the causative agent of one of the deadliest pandemics in human history [12,115,116,117,118]. Influenza virus sequences obtained from a formalin-fixed, ethanol-preserved North American wild goose collected in 1917, however, suggested that the 1918 HA segment was not directly acquired from birds, but had likely already circulated for some time in humans or other mammals [119]. Poliovirus (PV) RNA fragments were retrieved from fifty-year old formalin-fixed, paraffin-embedded spinal cord samples of Norwegian poliomyelitis fatalities dating to 1951–1952 [120]. Subsequent sequencing suggested that the outbreak could most likely be attributed to wild type PV1. A further RNA virus sequenced from fixed autopsy material is measles morbillivirus (MeV), one of the most infectious viruses known. As MeV infection induces life-long sterilizing immunity, the virus needs large groups of naïve hosts to sustain itself. Although an effective vaccine is available, measles continues to break out, as it did in the past. After retrieval of an almost complete MeV genome from a 1912 formalin-fixed lung specimen, Düx et al. calculated the entry of MeV, likely from a bovine precursor, into the human population as early as the 6th century BCE, a time period that saw the rise of big cities, supplying the virus with its required number of targets [121]. 

In recent years, RNA and DNA extraction from formalin- or alcohol-fixed samples, which has been much more challenging than nucleic acid isolation from frozen or fluid samples, as formaldehyde crosslinks nucleic acids and proteins, has been improved, potentially unlocking archival pathology collections for routine infectious diseases testing [122,123,124,125,126,127,128]. For an overview of ancient pathogen DNA recovered from the various biological sources, see Table 2.

### 3.5. Historical Publications as a Source of Past Epidemics

From the texts and inscriptions of early human civilizations up to contemporary scientific publications, ever since the invention of writing, people have documented evidence of disease and disaster. For instance, the ancient Greek tragedian Sophocles and historian and general Thucydides described the Plagues of Thebes (430–420 BCE) and Athens (430–426 BCE), respectively, while accounts of the Justinian Plague (541–543 CE) destroying the Roman empire, appeared in Arabic, Greek, Latin, and Syriac works [158]. Several ancient Egyptian “medical” papyri, such as the Ebers, Smith, and Hearst papyri, describe disease symptoms and epidemics that have been interpreted to refer to current pathogens [159]. (Critical) reviews of ancient epidemics in literature can be found in [159,160,161]. 

Outnumbering the ancient writings mentioning epidemic diseases are present papers speculating on the causative microorganisms from these accounts. However, not only are disease symptoms hardly diagnostic, translating or interpreting historical descriptions can be equally challenging. For instance, the Septuagint (LXX) and the Masoretic Text (MT), used in Old Testament translations, differ significantly as to the account of the plague of Ashdod (1190, or 1141 BCE), as described in Samuel 5:6–12. The coincident arrival of mice (or rats?) is not described in MT, although later on in both texts, five golden rats (or mice) must be provided as a sacrifice to free the town’s inhabitants, both young and old, from the plague [162]. The disease symptoms of that plague are nowadays translated as tumors, or boils, of the groin, which together with the rats would make an excellent case for bubonic plague as a putative cause (discussed in [163]). However, if those rats were mice, the case would be much weaker. Regarding pathology, the original texts mention “boils” or “sores” on the anus [162], which is quite unlike *Y. pestis* infection as we know it.

Descriptions of the Plague of Athens and the Justinian Plague inspired researchers to explore their actual causes. After analysis of three teeth samples from a mass burial pit at Kerameikos, Greece, the likely cause of the first was determined to be typhoid fever due to *Salmonella enterica* infection [11]. That claim was refuted by others, based on subsequent phylogenetic analysis of the initial results [164]. Teeth samples from individuals buried in Bavaria, Germany at the time of the Justinian pandemic, which affected large parts of Europe, were confirmed by two ancient DNA laboratories to contain DNA from an early, independent, lineage of *Yersinia pestis*, making it the first plague pandemic in history [52,144]. Ethiopian sources from the 13th–15th centuries also contain references to epidemics, possibly of plague, one of which wiped out so many people, that “no one was left to bury the death” [57,165]. Arabic, Egyptian, Syriac, and Sudanese texts likewise refer to epidemic diseases, which may or may not have been plague [57]. The 16th century Mexican “catastrophy” burials, which tested positive for *S. enterica* serovar Paratyphi C DNA, were backed up by evidence from Aztec painted manuscripts, the Codex Telleriano-Remensis and the Codex en Cruz, in which drawings of stacks of corpses and of figurines displaying symptoms of nose bleeds (epistaxis) and a body rash, respectively, are believed to represent an epidemic starting around 1544 in New Spain [75]. Indeed, epistaxis, and a so-called “rose spot” rash are clinical symptoms of enteric fever, although they are not the most prominent ones. Age, geography, and the specific causative organism are known to widely influence the signs and complications of enteric fever [166,167].

From around the 16th century onwards, parish registers in Europe kept track of baptisms, marriages, and deaths, and can be used as sources of unexpected or increased mortality in a neighborhood. Often, the churchyards related to the registers are known, and could be a source of ancient DNA, although many of them, and the skeletal remains, have been destroyed over time. Sometimes, records provide the cause of death, for instance, death registers from Bergen op Zoom, the Netherlands, provide this information from 1779 onwards. From that source, for instance, we learn that there was a particular severe outbreak of smallpox in the summer of 1790, with more than half of fatalities in July and August of that year being due to young children dying from it [168]. In the winter of 1797–1798, smallpox raged again, with an even higher mortality this time. In the autumn of 1807, there was a deadly epidemic of something called “nervous fever” (*Febris nervosa epidemica*), most likely enteric fever, to which about 45% of adult deaths were attributed. Interestingly, no casualties were seen in children. Likewise, from the 16th century onwards, medical doctors in Europe and the USA started to prepare lists and publications on health and disease from their own observations. Many 19th century publications were, for instance, devoted to cholera, then a new disease that was spreading around the world and causing deadly pandemics [169]. From medical publications listing the annual diseases prevalent in town and villages, it is obvious that in these times of poverty, crowding, malnutrition, and lack of vaccines, infectious diseases were common. A problem with the historical descriptions, however, is that the names of the maladies, or the symptoms listed, do not always ring a bell in our present times. *Rubeola*, *scarlatina,* and *febres intermittentes* likely indicate measles, scarlet fever, and malaria, respectively, but the medical conditions that would nowadays best describe *morbi inflammatorii pectoris* and *erysipelas intestinorum*, as given in 19th century publications, are difficult to ascertain [170]. In addition, biological samples connected to the reports are likely to be rare. An exception is the analysis of a wet intestinal sample derived from a 1849 victim of the second cholera pandemic in the USA, from which a complete genome of *Vibrio cholerae* has been reconstructed [112]. 

In general, historical publications and other written evidence can be helpful in tracing the pathology, sites, and samples of past epidemics, but as those texts are not easy to translate into current medical language, and there is usually no direct connection with human biological samples, there is only limited use for them in ancient pathogen research.

#### A Historical Enigma: Sweating Sickness of 15th and 16th Century Europe

An epidemic that came and went between 1485 and 1551 is the one given the name “English sweating sickness”, “sweating disease”, or “Sudor Anglicus” [171]. It first appearance was indeed in London, England, after the battle of Bosworth. Four more episodes followed in 1508, 1517, 1529, and 1551, of which the 1529 outbreak reached far into mainland Europe. The disease was also described in the Netherlands and Belgium in 1489 [171]. Between the major epidemics, cases were reported from several European countries [171]. Symptoms included high fever, headache, hyperhidrosis, palpitations, stomach ache, dyspnea, madness, and many more. The estimated mortality rate was 30–50% [171,172]. John Caius of Cambridge, then president of the Royal College of Physicians, published in 1552 an account of the sweating disease, the first monograph entirely devoted to an infectious disease [173]. Over the years, the causative agent of sweating disease has been proposed, and mostly dismissed, to be *Bacillus anthracis* (the cause of anthrax), *Vibrio cholerae*, influenza virus, or a hantavirus, amongst others [171,172,174,175,176,177]. Cheshire et al. have argued that the etiological agent of sweating disease would likely be a virus affecting the autonomic nervous system [172]. Interestingly, autonomic dysfunction, sometimes with hyperhidrosis, is a prominent feature of acute SARS-CoV-2 infection [178,179]. Rhabdoviruses, a virus family to which for instance rabies virus belongs, may likewise be a possibility to be considered. Bovine ephemeral fever virus (BEFV), an arthropod-borne rhabdovirus, for instance, induces rapid pulse and tachycardia accompanied by high fever and respiratory symptoms in cattle, commonly affecting adults over young animals, and fat bulls over lean steers [180,181]. Listlessness, paralysis, coma, and death may occur [180]. Little known rhabdoviruses, such as members of the genus tibrovirus, are increasingly being detected in humans [182,183]. Serological surveys in African populations have shown high exposure rates to rhabdoviruses [184], illustrating that such viruses have probably been spreading for some time. 

As for samples, John Caius describes in his monograph the death and burial in 1551 of the Duke of Suffolk, Henry Brandon, aged 15, and his brother Charles, aged 13–14, due to sweating disease. As their graves in Buckden, Cambridgeshire, England, are still there, the remains could possibly be subjected to ancient DNA analysis. However, current techniques are not yet optimized for ancient RNA analysis from bone or teeth [185], should sweating disease have been caused by an RNA virus. In contrast to most rhabdoviruses, tibroviruses are able to cause a viremia, allowing them in theory to be isolated from dental pulp. Another opportunity to define a cause for sweating disease could perhaps be found in victims of the “Picardy sweat” or “suette miliaire”, a disease with some resemblance to the English sweating disease, which caused 196 outbreaks in Europe between 1718 and 1874, when the illness similarly disappeared [177]. However, no samples belonging to Picardy sweat patients have yet been traced, although a few names of victims are known.

## 4. Discussion

Investigating epidemic pathogens of the past has already resulted in changing views on the history of infectious diseases and on the evolution of microbes. For instance, it has been confirmed that most of the “catastrophy” burials in Eurasia in the past 700 years or so were, indeed, due to fatal *Yersinia pestis* infections, making *Y. pestis* without doubt the most prominent epidemic pathogen in history, at least on that continent. A second important epidemic pathogen turned out to be *Salmonella enterica* serovar Paratyphi C and precursor lineages. Enteric fever epidemics due to S. Paratyphi C were seen in Bronze Age China, in medieval northern Europe, and in 16th century Mexico and Spain [74,76,77,78]. Whereas plague is now limited to around ~100–3000 cases per year, infections caused by S. Typhi and S. Paratyphy A, B, and C, the only four of the ~2600 *S. enterica* serovars able to infect systemically, are still a major problem in low income countries, with S. Typhi and S. Paratyphi A causing over 14 million cases annually [167,186]. The Para C lineage likely arose in Neolithic Europe from pig *Salmonellae* [79,187], and has thus been able to maintain its virulence in humans over millennia. It is, however, less prevalent at present, which could be partly due to host genetic selection, for instance at the HLA-DRB1 locus [188,189]. The secondary major historical pathogens, at least in Europe, proved to be louse-borne microbes, namely the agents of epidemic typhus, trench fever, relapsing fever, and possibly plague, which has been speculated to have also been transmitted by lice [71]. Louse-borne diseases essentially affect the poor, and thrive during war, crowding, and famine. In ancient DNA studies, trench fever-inducing *Bartonella quintana*, and typhus-inducing *Rickettsia prowazekii* were mainly found in association with the military, illustrating that the living conditions in garrisons and during army campaigns in history offered plenty of opportunities for lice to breed and spread. Both pathogens continue to cause problems to this day, as for instance exemplified by a 1997 large outbreak of epidemic typhus and trench fever in Burundi, a 1998 epidemic typhus outbreak in a Russian mental nursing home, and the high prevalence of *B. quintana* infections seen among the homeless in the USA [190,191,192]. 

Regarding the sources for the study of these historic epidemics, burial patterns have been proven to be highly informative to locate pathogens. Multiple burials, with signs of a crisis and without evidence of physical damage to the remains, have often been found to be associated with epidemic pathogens in Europe and Asia. The number of people per grave, from two to dozens, likely correlates with the local population density, so that a double burial can already indicate an epidemic disaster in a lowly populated area. The biological sources that are found in graves are usually limited to bones and teeth, implying that the pathogens involved would be those targeting bone, or present in blood. The pathogens listed with bone and dental pulp sources (Table 2), for instance, *Y. pestis* and *S. enterica* Paratyphi C, are indeed able to cause a bacteremia. However, the absence of sequences of these two bacterial species in one of the largest mass burial graves from 14th century Lübeck [77] suggests that another pathogen could have be involved in these epidemics; one that may not be present in blood at all. Looking at current pandemic pathogens, such as human immunodeficiency virus (HIV), Ebola virus (EBOV), and SARS-CoV-2, the first two viruses do target immune system cells, but the latter can hardly been found in blood, and would not likely be picked up should our descendants ever investigate the surplus of burials dating to 2020–2022 CE. Another problem with putative SARS-CoV-2, and EBOV, retrieval is that they are RNA viruses. Ancient RNA techniques, particularly when isolating RNA from sources other than “wet” tissue, are still being developed. However, remarkable results, including the sequences of RNA virus genomes from 750–1000-year-old grains and maize cobs, have already been published [185,193,194,195]. Human genomes from all over the world show strong selective sweeps, resulting from extensive adaptations to RNA virus infections, suggesting that this class of pathogens was as important in past epidemics as it is now [196]. Therefore, it is important for the study of (pre)historic epidemics that ancient RNA techniques are further developed, so that they can be reliably deployed. DNA viruses have left a much less significant imprint on the human genome [196]. In contrast to bone and teeth, so-called “wet” samples, either liquid, fixed or frozen, and either stored in freezers or in permafrost, should be able to deliver nucleic acids from any pathogen targeting that tissue or organ. Unfortunately, such samples are in general not much older than ~300 years, except for permafrost remains, and so cannot routinely be used to track epidemics from the distant past. 

Although burial patterns are a good predictor of where to find evidence for (pre)historic epidemics, and dental pulp is an excellent source of ancient DNA, the recovery of pathogen genomes is limited to those present in blood, or targeting bone. Samples of specific tissue or organs, which can be used to find other pathogens, are in general much younger, so that their use is limited. Collections of Egyptian canopy jars, containing mummified organs, or the dried organs present in some natural mummies, could be a putative source of DNA from pathogens with a tissue tropism other than blood or bone. However, the success in extracting DNA from such sources is generally lower, and RNA virus recovery is currently a challenge from most samples. Similarly, tracking pathogens in our genome may result in finding remnants of viruses that have the capacity to enter the germ line or the early embryo [197]. Those will mainly have DNA genomes and express integrases, in other words, they will chiefly be retroviruses. Any evidence of true RNA or non-integrating DNA viruses is fragmentary at best, and will hardly suffice to define prehistoric epidemics. For instance, fragments of borna- and filoviruses, but not complete genomes, are part of vertebrate genomes [25,198]. Such EVEs can only tell us that at the time of integration, there must have been plenty of borna- and filoviruses around, but details are hard to come by after so much time.

At present, burial patterns in relation to epidemics have mostly been investigated in Eurasia, but it is not unlikely that the increased detection of large settlements, for instance, the pre-Hispanic sites discovered in South and Middle America [199,200], will provide fertile new hunting grounds for pathogens. At one Amazonian site, 103 burials were recently discovered, though none of them were reported to show signs of emergency [200]. Nevertheless, large parts of the world are still uncharted territory with regard to (pre)historic epidemics. For instance, very little research has been done in Africa, Australia, and North America. More limitations apply when combining burials with pathogens, as for instance cultures that do not bury their dead cannot be investigated in this way. 

Historical documents can be used to trace places of interest, or be used to add details, but their clinical descriptions of diseases are in general too unspecific or too indecipherable to be of any real use. Furthermore, there are far more descriptions of epidemics in the last two millennia than there will ever be archeological sites. On the other hand, some sites with mass graves have already been discovered, but are still lacking a pathogen, such as the victims from an unknown epidemic in 4–5th century Florence, and 14th century Lübeck [77,96]. 

Paleopathology has also been used to guide ancient pathogen research, sometimes with surprising outcomes. For instance, a mid-16th century Italian child mummy with skin pustules suggestive of smallpox was found to contain not VARV, but HBV sequences. This finding implied that the child had died from another condition, possibly Gianotti-Crosti syndrome, although that is, as far as we know, a self-limiting malady [9]. In contrast, a 300-year old Siberian mummy did harbor VARV reads, but there was no evidence of skin lesions, while for the mummy of Egyptian pharaoh Ramses V, which displays extensive pustules, especially on its face and lower abdomen, no VARV has been found to date [80,159]. Therefore, relying on skin pathology, which in the recent past was always a clear sign of smallpox, has proven to be problematic when trying to connect pathology and pathogen in historic specimens. 

Next to providing a time and location, pathology descriptions are one of the main features of old epidemical accounts. However, because clinical symptoms are mostly too general to be diagnostic of a certain disease, and as the interpretation of historic writings is often debatable, those descriptions are only useful as additional information at best. Another drawback is that medical symptoms as a result of infection may change over time as the host and pathogen adapt, so that what we see now may not be identical to the historic disease expression. Similarly, patterns of mortality may change when immunity is retained in the survivors of a first wave. Infections may then turn into childhood diseases, a pattern seen for instance in later plague epidemics [48]. In contrast, older age is an important risk factor for mortality in coronavirus epidemics, such as COVID-19, where the middle aged and the elderly have died in much larger numbers than the younger generations [201,202]. Such a skew in demographics may be important to keep in mind when investigating burial sites. Interestingly, the English sweating disease was reported to have preferentially killed middle aged men, with few deaths observed in children, women, and the elderly [172,175]. 

A prerequisite for the advancement of our knowledge of ancient diseases is that collaborations between archeologists and molecular biologists should be increasingly sought and perpetuated, since, despite some struggles, both fields have much to gain from each other [203,204]. Digitizing medical and museum collections may also open up these sources of old pathogens to global researchers [118]. The current development of non-destructive techniques could play an important role in gaining access to these resources [205].

## Figures and Tables

**Table 1:** Sources available for the study of ancient pathogens and historical epidemics.

Source	What Can be Retrieved? ^1^	What is Difficult to Determine?	Level of Study
Contemporary genomic DNA	Germ line infiltrating (retro)viruses	Disease symptoms	Pathogen, transmission dynamics
	Prevalence of protective alleles	Pathogen	Indirect evidence of epidemic
Archeological and historical sites	Bone and teeth samples for DNA analysis; burial patterns may assist in selection	Pathogen, disease symptoms	Epidemic
Archeological and historical samples	Ancient DNA	Disease symptoms ^2^ Prevalence	Pathogen
Pathology collections	Ancient DNA and RNA	Prevalence	Pathogen
Historical publications	Outbreaks and disease symptoms	Pathogen identification	Epidemic

^1^ Approximate age of the infection can be estimated from all sources ^2^ Bone or skin lesions due to chronic *Mycobacterium* or *Treponema* infection, however, can reveal pathology. Chronic infections with *Plasmodium* spp. may induce hyperostotic skull lesions (“pitting”), which are, however, not diagnostic.

**Table 2:** Ancient pathogen nucleic acid retrieved from human sources.

Biological Source	Pathogen Characteristic(s)	Ancient Pathogen Detected	Reference
Bone	Blood-borne Targeting bone *	HBV	[9,15]
		*Mycobacterium leprae*	[59,60,62]
		*Mycobacterium tuberculosis*	[63,64,65,67]
		Parvovirus B19	[91]
		*Salmonella enterica* Paratyphi C	[79]
		*Treponema pallidum*	[83,84,129]
		*Yersinia pestis*	[130]
		VARV	[89]
Dried tissue, such as mummified skin or organs	Tissue tropism	*Helicobacter pylori*	[131,132]
		HBV	[9]
		Human papilloma virus, HPV	[133]
		*Mycobacterium tuberculosis*	[66,134,135]
		VARV	[136,137]
Frozen/fixed—wet—tissue, incl. blood	Tissue tropism	HCV	[138]
		*Helicobacter pylori*	[139,140]
		HIV	[113,114]
		Influenza virus	[115,116,118]
		Measles virus	[121]
		Poliovirus	[120]
		VARV	[80]
		*Vibrio cholerae*	[112]
Tooth, dental pulp	Blood-borne	*Bartonella quintana*	[68,69,70,141,142]
		*Bordetella pertussis*	[143]
		*Borrelia recurrentis*	[72]
		*Haemophilus influenzae*	[81]
		HBV	[14,15]
		*Mycobacterium leprae*	[59,60]
		*Mycobacterium tuberculosis*	[65]
		Parvovirus B19	[91,92]
		*Rickettsia prowazekii*	[68,69]
		*Salmonella enterica* Paratyphi C	[74,76,77,79]
		*Treponema pallidum* spp.	[84]
		*Yersinia pestis*	[10,48,51,52,54,144]
		VARV	[89]
Tooth, dental calculus	Presence in oral cavity	*Mycobacterium leprae*	[145]
		Oral microbiome, including pathogens	[102,103,104,107,146,147,148,149]
Paleofeces	Tropism for gastro-intestinal tract	Intestinal microbiome, including pathogens	[101,103,105,106,108,146,150,151,152,153]
		Helminths	[93,94]
Other: Birch pitch	Presence in the oral cavity/expression in saliva	Epstein–Barr virus, EBV (human gammaherpesvirus 4)	[154]
Other: Calcified nodules	Presence in genitourinary tissue	*Gardnerella vaginalis* *Staphylococcus saprophyticus*	[155]
	Presence in abdominal/pelvic tissue	*Brucella melitensis*	[156]

* Blood-borne pathogens can also end up in bone, where nucleic acids adsorb to, and are protected by, apatite crystals composing the mineral part of bones (and teeth) [157].
